# Changes in stride pattern of elite women's 400 metres hurdles from 2019 to 2022: an analysis by performance level

**DOI:** 10.3389/fspor.2025.1515441

**Published:** 2025-02-27

**Authors:** Nerea Casal-García, José Luis López-Del Amo

**Affiliations:** ^1^ Institut Nacional d'Educació Física de Catalunya (INEFC), Universitat de Lleida (UdL), La Seu d'Urgell, Espanya; ^2^ Institut Nacional d'Educació Física de Catalunya (INEFC), Universitat de Barcelona (UB), Barcelona, Espanya

**Keywords:** athletics, observational analysis, pacing strategy, 400 metres hurdles, stride pattern

## Abstract

**Introduction:**

The main objective of this study was to investigate the stride patterns of elite women's 400 m hurdles which have undergone significant evolution in recent years, resulting in a complete change in the stride patterns of the athletes.

**Methods:**

Two hundred and fifty-five individual performances of all participants in the World Championships in Doha 2019 and Oregon 2022, the Tokyo 2020 Olympic Games and the 2022 European Championships were analysed based on video recordings taken during the competition. The entire sample was divided into 3 performance-level groups (higher performance, intermediate performance, and lower performance). Correlation analysis, analysis of variance (Kruskall-Wallis) and linear multiple regression were performed.

**Results and discussion:**

A total of 18 stride patterns have been identified, of which the most used by the highest performing athletes (58%) was the *n* + 1 pattern (consisting of adding a step between hurdle's intervals) maintaining 15 steps up to the 8th hurdle and then switching to 16 steps between hurdles 8th and 10th. In contrast, the majority of LP athletes used an *n* + 1 + 1 strategy, transitioning from 15 to 16 steps between the 5th and 6th hurdles (59%) and further increasing to 17 steps between hurdles 7th and 10th. Moreover, high-performance athletes take fewer strides during the race, with significant differences observed across all race intervals, particularly in the second half of the race. The number of steps between hurdles 8 and 9 predicted 78% of the *Δ*R_2_ of the athlete's total number of steps during the race, highlighting the importance of stride pattern and its changes during the final phase of the race.

## 1 Introduction

The women's 400 metres hurdles (400 mH) has evolved later than the male discipline due to their delayed official recognition and inclusion in major national and international championships. This event is considered highly demanding, requiring not only motor skills like speed and strength, but also the technique of clearing hurdles (1).

Traditionally, studies on 400 mH have focused on two essential parameters: stride pattern and effort distribution. However, these studies are still scarce and have faced various methodological problems, such as limited sample sizes (only including finals in major championships) (1–3) or data analysis based on television footage, which results in missing information due to zooming and switching between camera angles (3–6). Moreover, in recent years, the event has seen an important evolution in both men's and women's categories, with world records being broken for both. Not only have these records improved, but the average performance has also increased considerably (1, 4). Despite this, to the best of our knowledge, there are limited studies examining the evolution of this period (2019–2022), with only one study including data from 2019 (5).

The 400 mH effort distribution has been studied from different perspectives. The times for the two halves of the race and the differences between them have been described and analysed (7). Since the race has 10 evenly spaced hurdles (35 m apart), it can be divided into 9 intervals between hurdles (8).The race has also been divided into three equal sections of 105 m, considering the intervals between hurdles (H1-H4, H4-H7, H7-H10) (9), and three types of athletes have been defined based on the time distribution in these sections (“speed”, “technical”, and “endurance”) (5, 9, 10). Regardless of the athlete's performance level, the 400 mH race can be divided into three phases: an initial acceleration phase (up to the 2th or 3th hurdle), a relative maintenance of speed (between the 2th and 4th hurdle), and a sustained loss of speed (from the 3th or 4th hurdle to the 10th) (9). Since running economy is fundamental in this event, touchdown times must also be addressed, as they are crucial after each hurdle and particularly after the last hurdle to return to sprint form (11).

Stride pattern is a fundamental element in the analysis of 400 mH. This pattern is determined by the number of steps taken between hurdles (12). The hurdle rhythm is defined as running with the minimum loss of speed, regardless of the fatigue and clearing the subsequent hurdle (1, 7, 13). Maintaining a stride pattern requires proper physical preparation due to speed loss caused by fatigue during the race (14–17). The race strategy caused by fatigue should also be understood through studies on the 400 m flat, an event with a higher anaerobic component but sharing characteristics of effort distribution with the hurdle event (15, 18–21). This highlights the importance of the ability to maintain an optimal stride pattern throughout the race as a fundamental training goal in the 400 mH (9).

In the women's event, the steps between hurdles are typically between 15 and 17 (13, 22). However, the number of steps has decreased in recent years (1, 4). Fatigue during the race can increase the number of steps and four basic structures (stride patterns) have been identified (22): n, where the athlete starts and finishes with the same number of steps; *n* + 1, where one step is added; *n* + 1 + 1, where one step is added twice; and *n* + 2, where two steps are added. The most common stride pattern among high-level female athletes is *n* + 1 + 1, but top athletes also used the *n* + 1 pattern (6, 22). However, these structures do not account for the changes in stride patterns that occur with one fewer step. This stride pattern has been considered incorrect (7), but in recent years it has been observed that elite male athletes use it, including some of the best athletes in recent major Championships. Stride patterns can be influenced not only by increasing or decreasing steps during the race but also by the selection of the lead leg and the athlete's anthropometric characteristics. In addition to athlete's preferences for their lead leg, attacking with the left leg on curves is preferable. This allows running closer to the curve's inner lane and minimizing the likelihood of the left leg trailing below the hurdle height, which could result in disqualification (22).Furthermore, anthropometric factors influence performance, as taller athletes benefit from easier hurdle clearance and a reduced number of strides during the race, both of which are linked to improved performance. However, the anthropometric characteristics of elite athletes vary considerably among individuals (13, 23).

The most critical part of the race is the second half, as it is where the greatest differences between high-level and lower-level athletes have been observed (4–6). Elite athletes experience less loss of speed towards the end, which can result in a reduced need for changes in stride patterns. Furthermore, the stride length in the second half of the race seems to be crucial for performance (24), also being of great importance in the 400 meters flat (25). However, the reduction in the number of strides should not be forced; rather, it should be a consequence of the enhancement in performance, physical conditioning, and adaptation to the athlete's characteristics (5). An excessive increase in stride length, beyond the athlete's capabilities, may lead to a premature fatigue, forcing an early rhythmic change and further intensifying fatigue.

Therefore, the main objective of this study was to analyse the changes in stride patterns of elite female 400 mH athletes. A secondary objective was to analyse the relationship between the number of steps performed during the race and the final result, as well as the differences between groups. It was hypothesised that a lower number of steps would be correlated to race time (7). A third objective was to examine how these relationships may be related with stride pattern changes. It was hypothesised that female athletes usually add one stride between hurdles twice in a race (*n* + 1 + 1), starting with 15 steps, changing on the second curve (between hurdles five and eight) (5). Furthermore, we hypothesised that the improved performance in the last years could have led to changes in the athlete's stride patterns and using a linear multiple regression model could help improve the analysis of the stride pattern and key performance factors.

## 2 Material and methods

### 2.1 Participants

Two hundred and fifty-five individual performances in the women's 400 mH were considered (mean ± SD age 26.2 ± 3.8 years), including all participants from 4 major championships (World Championships in Doha 2019 and Oregon 2022, Tokyo 2020 Olympic Games, and European Championships in Munich 2022). A total of 18 heats (*n* = 130), 12 semifinals (*n* = 94), and 4 finals (*n* = 31) were included. Within the sample there are found 3 previous world records. Data from athletes who did not finish the race, were disqualified, or did not complete the race competitively were excluded according to inclusion criteria. All participants completed between 1 and 10 races, depending on their qualification for subsequent rounds. In the European Championships, the top 12 ranked athletes in the continental ranking bypassed the heats and advanced directly to the semifinals. The average final time was 55.48 ± 1.42 s.

### 2.2 Design and data collection

**Figure 1:**
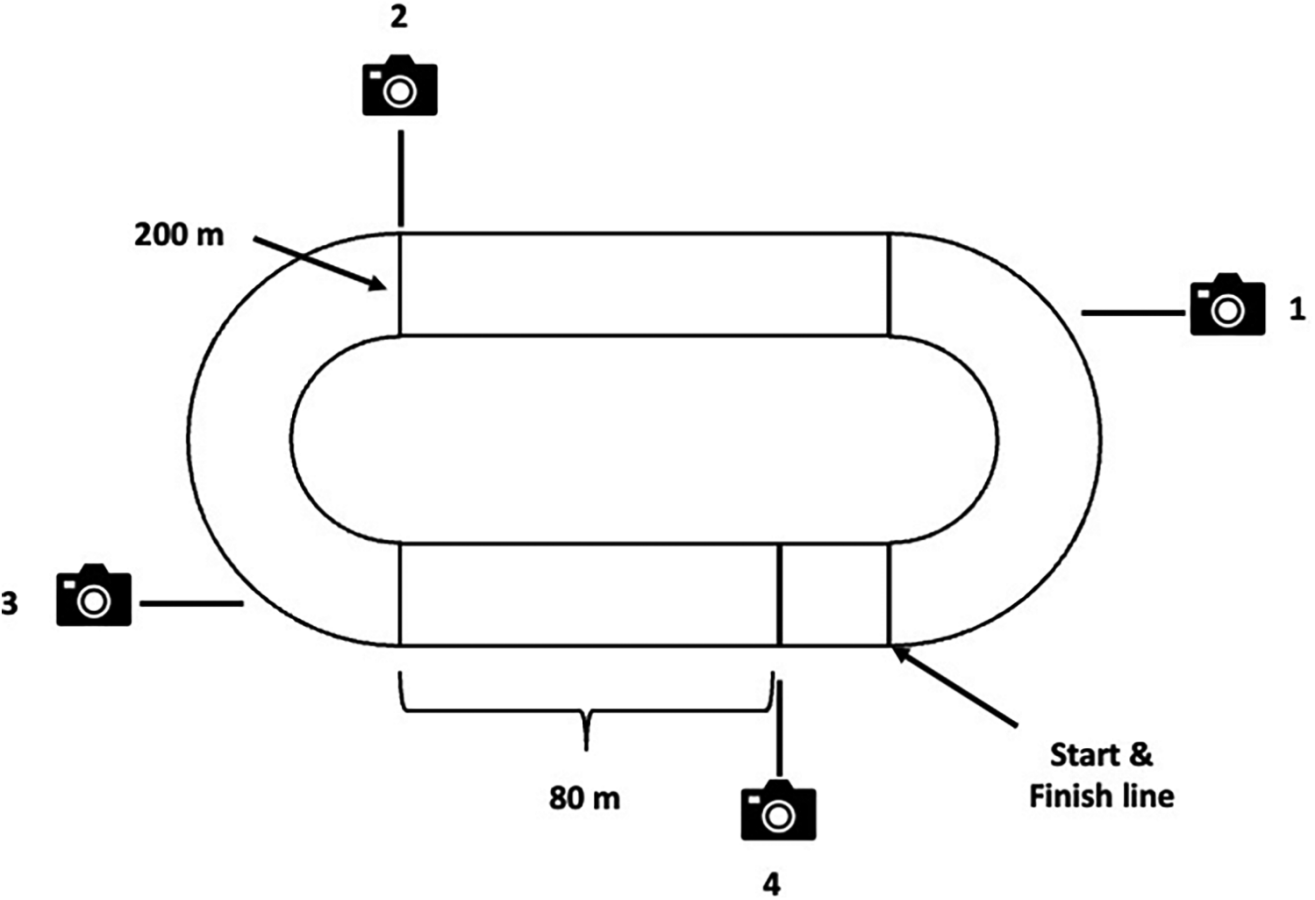
Camera**’**s location, start line (lane 1), 200 m mark (lane 5) and finish lane.

All races were filmed with Sony R6 cameras in high definition at 60 Hz from the stadium stands (Figure 1). One camera was placed at the end of each curve, another at the 200 m mark, and the last one 20 m from the finish line on the main straight. All cameras kept all the athletes in frame to facilitate later analysis and used the official's start signal as a time reference. The footage was analysed using Kinovea software (v0.9.5), a reliable method for analysing spatial and temporal variables (26). The data was obtained by 3 independent experts. Due to the nature of the data, if there were inter-observer differences, the recordings were reviewed again.

The sample was divided into three groups based on official final times: higher performance (HP, 50.68–54.91 s), intermediate performance (IP, 54.93–55.93 s), and lower performance (LP, 55.94–60.36 s). When dividing the sample into equal-sized groups, athletes with the same 400 mH time were classified in the same group. This ensured that identical performances did not appear in different groups. The study was approved by the Ethics Committee of the Consell Català de l’Esport (026/CEICGC/2021) and no informed consent was needed due the observational methodology. To ensure anonymity, the database was encoded based on the official final result, so that no individual performance could be identified. All videos were recorded from the stadium's public area for the purpose of data collection.

### 2.3 Variable selection

The variables included in the study are related to the stride pattern (number of steps between hurdles intervals, number of steps from start to first hurdle, number of steps from the 10th hurdle to finish, total number of steps, number of steps in the first and second 200 m, location of the first stride pattern change *n* + X and total number of stride pattern changes during the race); and the official final time as a measure of performance level. The final step was counted as either a half step or a full step, depending on the position of the athlete's foot relative to the finish line. The stride pattern is determined by the number of steps in each interval and the changes in them that an athlete performs during the race (2, 27).

### 2.4 Statistical analysis

Descriptive statistics were performed for the entire sample and every group using mean, standard deviation, minimum, and maximum. For the number of strides between hurdles (discrete data), median, 25th and 75th percentiles, minimum, and maximum were used. The Kolmogorov-Smirnov test was used to analyse the normality of the variables due to group size. Frequency tables were used to show the distribution of change typologies (1st objective of the study). To assess the relationship between final time and independent variables, a Pearson correlation coefficient analysis was used for continuous variables and a Spearman rank correlation analysis for discrete variables. Moreover, a one-way ANOVA analysis or a Kruskal-Wallis analysis were performed to analyse the groups difference in continuous and discrete variables, respectively. The Levene test was performed to verify homogeneity. Bonferroni and Dunn *post hoc* tests (2nd objective of the study). Regarding rhythmic changes, the same procedure (3rd objective of the study). Thresholds for the interpretation of correlation coefficients were set and interpreted as follows (28): trivial (<0.1), small (0.1–0.29), moderate (0.3–0.49), strong (0.5–0.69), very strong (0.7–0.89) and near perfect to perfect (0.9–1.0). Cohen's d threshold was set and interpreted as follows (29): trivial (0–0.2), small (0.2–0.49), moderate (0.5–0.79), large (0.8–1.19) and very large (>1.2);Rank biserial threshold was established and interpreted as follow (28): small (0.1), moderate (0.1–0.3), large (0.5), very large (0.7), extremely large (0.9); and *η*^2^ thresholds were set as (30): trivial (<0.01), small (≥0.01), moderate (≥0.06) and large (≥0.14). Finally, a stepwise backward multiple linear regression was performed between the number of steps in each interval and the total number of steps, to answer our second hypothesis. Significance level was set at *p* < .05 and all data was analysed using JASP (0.18.3, University of Amsterdam).

## 3 Results

Frequency tables for all the stride patterns are shown in Table 1. Eighteen different stride patterns were found. The median of different stride patterns used per athlete was 2. Nineteen athletes (23%) maintained the same stride pattern throughout all the analyzed races. Only athletes in the HP group used a *n* pattern (*n* = 5, 6%), which means that they maintained the same number of steps throughout the race. The most often stride pattern in the sample is *n* + 1 + 1 (*n* = 126, 49%), which is used for nearly half of the sample. However, the majority of athletes from HP group used an *n* + 1 pattern (*n* = 49, 58%), being the second most used typology of changes overall (*n* = 80, 31%). Regarding stride patterns that allow athletes to maintain the same lead leg during hurdle clearance while increasing the number of strides, only the *n* + 2 pattern was observed, with limited use (*n* = 6, 2%).

**Table 1:** Stride patterns.

Stride patterns	LP (*n* = 86)	IP (*n* = 85)	HP (*n* = 84)	Overall (*n* = 255)
*n*	–	–	5 (6.0%)	5 (2.0%)
*n* + 1	7 (8.1%)	24 (28.2%)	49 (58.3%)	80 (31.4%)
*n* + 1 + 1	53 (61.6%)	48 (56.5%)	25 (29.8%)	126 (49.4%)
*n* + 1 + 1 + 1	11 (12.8%)	3 (3.5%)	–	14 (5.5%)
*n* + 1 + 1 + 1 + 1	2 (2.3%)	–	–	2 (0.8%)
*n* + 1 + 1 + 2	2 (2.3%)	–	–	2 (0.8%)
*n* + 1 + 1-1	–	2 (2.4%)	1 (1.2%)	3 (1.2%)
*n* + 1 + 1-1 + 1	1 (1.2%)	–	–	1 (0.4%)
*n* + 1 + 2	1 (1.2%)	1 (1.2%)	–	2 (0.8%)
*n* + 1 + 2-1	1 (1.2%)	–	–	1 (0.4%)
*n* + 1 + 2-1 + 1	1 (1.2%)	–	–	1 (0.4%)
*n* + 1-1 + 1	–	–	2 (2.4%)	2 (0.8%)
*n* + 2	1 (1.2%)	4 (4.7%)	1 (1.2%)	6 (2.4%)
*n* + 2 + 1	1 (1.2%)	1 (1.2%)	–	2 (0.8%)
*n* + 2-1 + 1	–	1 (1.2%)	–	1 (0.4%)
n-1 + 1		–	1 (1.2%)	1 (0.4%)
n-1 + 1 + 1	5 (5.8%)	–	–	5 (2.0%)
n-1 + 1 + 1 + 1	–	1(1.2%)	–	1(0.4%)

LP, lower performance; IP, intermediate performance; HP, higher performance.

Descriptive statistics and the correlation coefficient between the dependent variable (400 mH time) and independent variables [total number of steps in the race and each in each 200 m interval, location of the first stride pattern change (*n* + X) and the number of stride pattern changes (*n* + X) during the race] are presented in Table 2. All variables were correlated with the 400 mH time (*p* < .001). The steps during the whole race and in the second 200 m segment were strongly correlated (r = 0.67, *p* < .001). Steps performed during the first 200 m segment were strongly correlated (0.55, *p* < .001). Regarding the stride pattern changes, the location of the first *n* + X change was strongly correlated (*ρ*=0.54, *p* < .001) and the number of *n* + X changes showed a moderate inverse correlation with 400 mH time (*ρ*=0.40, *p* < .001).

**Table 2:** Differences between groups in final time (s), total number of steps (*n*), number of steps in each 200 m interval (n) and number and location of *n* + X stride pattern changes (n).

	Group						Overall, (*n* = 255)		
	LP (*n* = 86)		IP (*n* = 85)		HP (*n* = 84)				
	Mean ± SD	Min/max	Mean ± SD	Min/max	Mean ± SD	Min/max	Mean ± SD	r [95% CI]	*p*-value
Final time (s)	56.98 ± 0.83	55.94/60.36	55.42 ± 0.30	54.93/55.93	54.00 ± 0.88	50.68/54.91	55.48 ± 1.42		
Total number of steps (*n*)	198.6 ± 5.0	188.5/212.0	193.8 ± 6.0	180.5/207.0	188.4 ± 4.6	179.5/200	193.7 ± 6.7	0.67 [0.60, 0.73]	<.001
Total number of steps in the first 200 m (*n*)	96.4 ± 2.4	93.0/102.5	94.4 ± 2.9	87.5/101.0	92.8 ± 2.4	87.0/98.0	94.5 ± 3.0	0.55 [0.45, 0.63]	<.001
Total number of steps in second 200 m (*n*)	102.3 ± 3.3	94.8/112.0	99.4 ± 3.4	92.5/107.0	95.7 ± 2.7	90.0/103.0	99.1 ± 4.2	0.67 [0.60, 0.74]	<.001
Number of *n* + X changes (*n*)	1.3 ± 0.6	1.0/4.0	1.7 ± 0.5	1.0/3.0	2.1 ± 0.6	0.0/2.0	1.7 ± 0.7	−0.30 [−0.40, −0.18]	<.001
Location of the first *n* + X change (*n*)	6.3 ± 1.7	2.0/8.0	5.8 ± 1.4	2.0/9.0	5.1 ± 1.4	2.0/9.0	5.7 ± 1,6	0.54 [0.44, 0.62]	<.001

LP, lower performance; IP, intermediate performance; HP, higher performance; Min, minimum; Max, maximum; SD, standard deviation; CI, confident interval. Time variables have 2 decimals for optimal precision.

**Figure 2:**
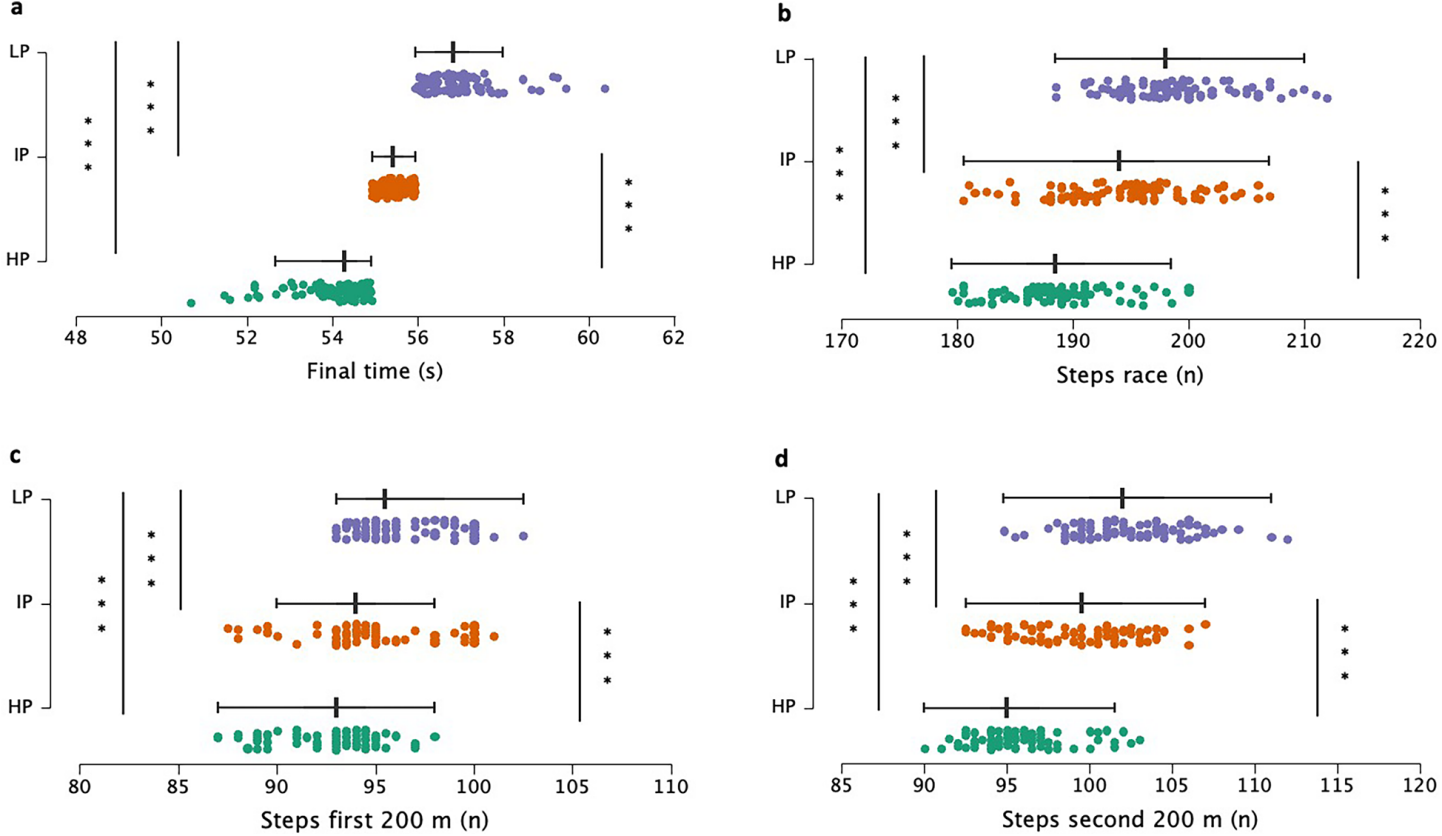
Group differences and distribution of step-related variables. Each point corresponds to an athlete. The variables shown are: **(a)** total time; **(b)** total number of steps during the race; **(c)** total number of steps in the first half of the race; **(d)** total number of steps in the second half of the race. LP, lower performance; IP, intermediate performance; HP, higher performance; *Statistically different (*p* < .05); **Statistically different (*p* < .01); ***Statistically different (*p* < .001).

Regarding the groups, there were differences between all groups, as shown in Figure 2. Regarding the total number of steps performed, significant differences were found (F(_2,252_^)^ = 81.34; *p* < .001, *η*^2^ = 0.39, large). The HP athletes did fewer steps during the race with respect to IP (*p* < .001, d = −1.03, large) and LP (*p* < .001, d = −1.96, very large). IP also exhibited fewer steps needed to complete the race compared to LP (*p* < .001, d = −0.93, large). Regarding the steps in each half of the race, differences were found in the first 200 m interval (F_(2,252)_ = 41.06; *p* < .001, *η*^2^ = 0.25, large). The HP groups took fewer steps with respect to IP (*p* < .001, d = −0.63, moderate) and LP (*p* < .001, d = −1.39, very large). There were also differences between IP and LP (*p* < .001, d = −0.76, moderate). Regarding the second half of the race, higher differences were found between the groups (F(_2,252_) = 94.92; *p* < .001, *η*^2^ = 0.43, large). The HP group took significantly fewer steps than IP (*p* < .001, d = −1.19, large) and LP (*p* < .001, d = −2.11, very large).

Significant differences were found between the HP, IP and LP groups in all variables related to steps in each interval (Table 3). Athletes in the HP group take fewer steps in all segments of the race compared to the other groups. The largest differences were found in the latter half of the race, although there are large differences between the HP and LP groups in all sections. Between H8-H9 great differences were found (*χ*^2^_(2)_ = 99.33; *p* < .001; *η*^2^ = 0.37, large), especially between HP and IP (*p* < .001, r_s_ = −0.62, very large). The differences became more accentuated in H9-H10 interval (*χ*^2^_(2)_ = 121.96, *p* < .001, *η*^2^ = 0.44, large) and in the H10-finish interval (F_(2,252)_ = 71.19; *p* < .001, *η*^2^ = 0.35, large). The differences between HP and LP were great in both intervals (*p* < .001, r_s_ = −0.67, very large; *p* < .001, d = −1.76, very large, respectively).

**Table 3:** Median, interquartile ranges (P25 and P75), and ANOVA between the number of steps in each interval by group (*n*).

Variable	Group			Overall, (*n* = 255)	Group differences	Post-hoc	Effect size
	LP (*n* = 86)	IP (*n* = 85)	HP (*n* = 84)				
Steps from start to H1	23 [23,24]	22 [22,23]	22 [22,23]	23 [22,23]	*χ*^2^ = 55.94	LP-IP*	−0.20
					*p* < 0.001	LP-HP***	−0.57
					*η*^2^ = 0.19	IP-HP***	−0.37
Steps H1-H2 (*n*)	15 [15,16]	15 [15,15]	15 [15,15]	15 [15,15]	χ^2^ = 40.91	LP-IP**	−0.22
					*p* < 0.001	LP-HP***	−0.40
					η^2^ = 0.16	IP-HP***	−0.18
Steps to H2-H3 (*n*)	15 [15,16]	15 [15,15]	15 [15,15]	15 [15,15]	χ^2^ = 45.90	LP-IP**	−0.24
					*p* < 0.001	LP-HP***	−0.42
					η^2^ = 0.18	IP-HP***	−0.18
Steps to H3-H4 (*n*)	15 [15,16]	15 [15,15]	15 [15,15]	15 [15,15]	χ^2^ = 35.36	LP-IP**	−0.19
					*p* < 0.001	LP-HP***	−0.37
					η^2^ = 0.14	IP-HP**	−0.18
Steps to H4-H5 (*n*)	15,5 [15,16]	15 [15,15]	15 [15,15]	15 [15,16]	χ^2^ = 52.08	LP-IP***	−0.17
					*p* < 0.001	LP-HP***	−0.45
					η^2^ = 0.20	IP-HP*	−0.27
Steps to H5-H6 (*n*)	16 [16,16]	16 [15,16]	15 [15,15]	15 [15,16]	χ^2^ = 69.54	LP-IP**	−0.31
					*p* < 0.001	LP-HP***	−0.52
					η^2^ = 0.25	IP-HP***	−0.20
Steps to H6-H7 (*n*)	16 [16,17]	16 [15,16]	15 [15,15]	16 [15,16]	χ^2^ = 82.24	LP-IP***	−0.30
					*p* < 0.001	LP-HP***	−0.57
					η^2^ = 0.31	IP-HP***	−0.26
Steps to H7-H8 (*n*)	17 [16,17]	16 [16,17]	15 [15,16]	16 [16,17]	χ^2^ = 85.09	LP-IP***	−0.34
					*p* < 0.001	LP-HP***	−0.57
					η^2^ = 0.33	IP-HP***	−0.23
Steps to H8-H9 (*n*)	17 [17,17]	17 [16,17]	16 [16,16]	17 [16,17]	χ^2^ = 99.33	LP-IP***	−0.35
					*p* < 0.001	LP-HP***	−0.62
					η^2^ = 0.37	IP-HP***	−0.27
Steps to H9-H10 (*n*)	17 [17,18]	17 [16,17]	16 [16,16]	17 [16,17]	χ^2^ = 121.96	LP-IP***	−0.39
					*p* < 0.001	LP-HP***	−0.69
					η^2^ = 0.44	IP-HP***	−0.30
Steps from H10 to finish lane	21 [20,21.5]	20 [19.5–21]	19 [19–20]	20[19.5,21]	χ^2^ = 89.36	LP-IP***	−0.43
					*p* < 0.001	LP-HP***	−0.72
					η^2^ = 0.35	IP-HP***	−0.29

LP, lower performance; IP, intermediate performance; HP, higher performance; H, hurdle.

* Statistically different (*p* < .05).

** Statistically different (*p* < .01).

*** Statistically different (*p* < .001).

To better understand the contribution of the number of steps in each interval to the 400 mH total number of steps, a stepwise multiple linear regression was performed, including the steps taken between all hurdles, as well as from the start to the first hurdle and from the last hurdle to the finish line, to improve the model's accuracy (Table 4). The model explains 98% of the variance in the total number of steps in the 400 m race, where the number of steps in the H8-H9 segment explained 78% of the variance in the final performance of the model and the steps between H2-H3 the 11% of this variance.

**Table 4:** Linear multiple regression about the influence of number of strides in each interval (*n*) and total number of strides (*n*).

							Collinearity		Parameters	
Model		No standardised	SE	Standardised	t	*p*	Tolerance	VIF	*Δ*R^2^	R^2^
6	Intercept	15.377	2.338		6.577	<.001				0.98
	Steps H8-H9	2.724	0.165	0.329	16.546	<.001	0.355	2.816	0,78	
	Steps H2-H3	4.184	0.202	0.332	20.700	<.001	0.543	1.841	0,11	
	Steps H5-H6	2.705	0.176	0.270	15.366	<.001	0.454	2.200	0,05	
	Steps H10-Finish	1.410	0.116	0.219	12.144	<.001	0.432	2.317	0,02	
	Steps Start-H1	1.203	0.088	0.167	13.610	<.001	0.538	1.860	0.02	

H, hurdle; SE, standard error; VIF, variance inflation factor; *Δ*, delta.

**Figure 3:**
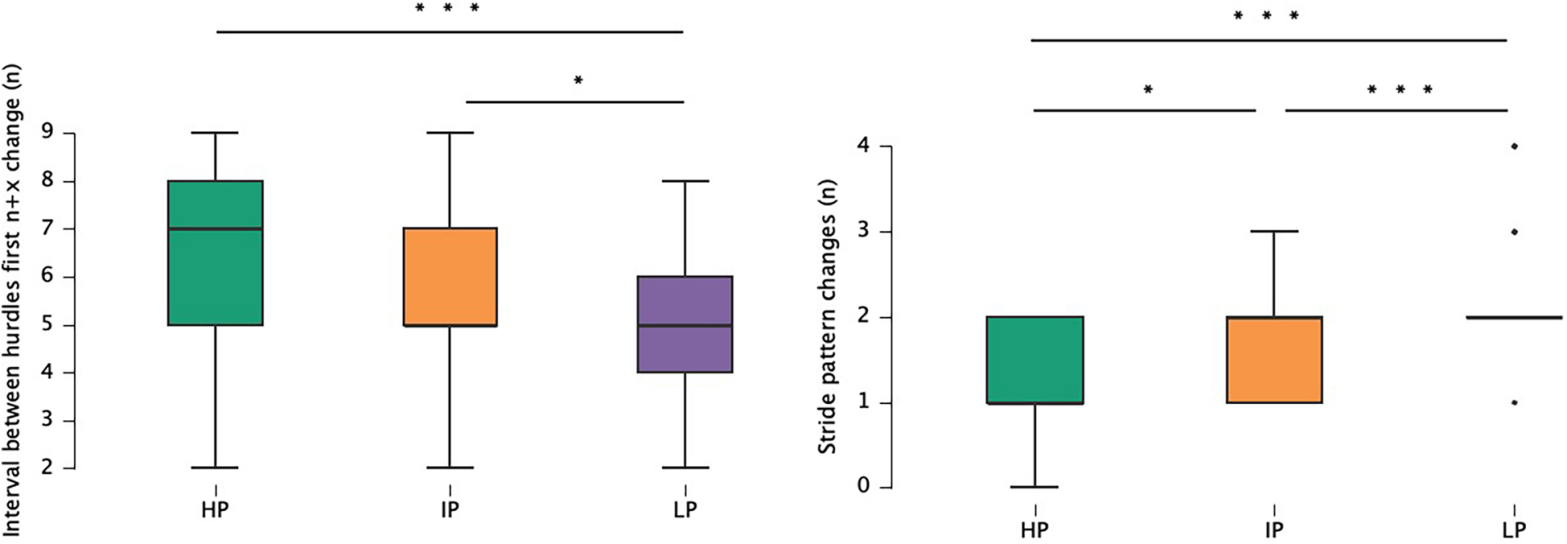
Group differences and distribution of variables related to stride pattern changes. Each point corresponds to an athlete. LP, lower performance; IP: intermediate performance; HP, higher performance; *Statistically different (*p* < .05); **Statistically different (*p* < .01); ***Statistically different (*p* < .001).

Stride patterns related to the third objective of the study, with differences between groups, are shown in Figure 3. There were differences between the number of changes performed during the race (*χ*^2^_(2)_ = 73.84; *p* < .001; *η*^2^ = 0.29, large). Furthermore, HP made fewer stride pattern changes than IP (*p* < .001, r_s_ = −0.28, small) and LP (*p* < .001, r_s_ = −0.54, large). Regarding the location of the first stride pattern change (*n* + X), there were differences between groups (*χ*^2^_(2)_ = 22.90; *p* < .001; *η*^2^ = 0.09, moderate), showing greater differences between HP and LP (*p* < .001, r_s_ = 0.30, moderate). Most athletes who performed a *n* + 1 pattern, added an step between hurdles 7th and 8th (31%) 8th and 9th (34%). In contrast, the athletes who made two changes advanced the first one, performing it between hurdles 5th and 6th (41%) and 6th and 7th (21%).

## 4 Discussion

The aim of this study was to analyse the stride pattern changes used by elite female athletes in 400 mH. among which the *n* + 1 and *n* + 1 + 1 patterns were the most used across the entire sample. Among the identified stride patterns, there are also some that involve the addition of two steps, either combined or not with the addition of another step, and even structures in which the number of steps is reduced and later increased again, with a maximum of four changes. Higher-performing athletes tend to take fewer strides during the race, either by adding fewer steps during the race or by taking fewer steps in the first interval (between hurdles 1 and 2), or from the start to the first hurdle. Among the HP athletes, the majority employed an *n* + 1 strategy, with 56% maintaining 15 steps up to the 8th hurdle before transitioning to 16 steps in the last two intervals (8th to 10th hurdles). In contrast, most LP athletes adopted an *n* + 1 + 1 strategy, shifting from 15 to 16 steps between the 5th and 6th hurdles (59%) and further increasing to 17 steps in the final three intervals (7th to 10th hurdles). To the first hurdle, 56% of HP athletes took 22 steps. In the IP group, 40% completed 22 steps and 35% used 23 steps. Among LP athletes, 56% employed 23 steps. From the last hurdle to the finish, the majority of HP athletes took between 19 and 20 steps (77%), IP athletes between 19 and 21 steps (87%), and LP athletes between 20 and 22 steps (87%).

The number of steps during the race, especially in the second half, has a strong correlation with the 400 mH time, as found in previous studies (6, 24). However, this does not imply that reducing the number of steps will necessarily improve performance. The stride pattern should be adapted to the athletes, not the other way around (7). This suggests that performance improvements lead to a reduction in steps. On recent years athletes tend to start faster and with fewer steps but had a greater increase in steps in the final intervals (between H8-H10) (1). Data show that athletes in the HP group currently take fewer steps during the race, maintaining a 15 steps rhythm for more intervals between hurdles. However, a relationship between fewer steps and body height was found, making it essential to adapt the strategy to individual characteristics (13).

The stride pattern should not be understood only as the number of steps between each interval but should also consider other factors such as the increase in steps, the number of stride pattern changes, and their location (3). The first stride pattern change is typically located between the 6th and 8th rhythmic units (3, 6). This is due to the fatigue induced by race, which reduces stride length, optimising the running economy (13). However, the number of strides may vary among athletes and can be influenced by factors such as height, weight, and body mass index (BMI). In a sample of elite male athletes, taller individuals often preferred an *n* + 1 patterns, while shorter athletes tended to use an *n* + 1 + 1 pattern (13). According to Martín (30), the race can be divided into four phases: a first alactic phase up to the 5th hurdle at most; an alactic-lactic transition phase between the 5th and 7th hurdles; the third anaerobic glycolysis phase at the 8th hurdle; and the last phase in the straightaway, at the 9th and 10th hurdles. Therefore, a rhythmic change before the 5th hurdle might be considered a tactical error, as the athlete would not be prepared to execute the planned number of steps, resulting in an early performance loss (7). However, we observed that a small number of athletes in the sample made a change between 2th and 3th hurdles (11%), with one of them belonging to the HP group, which could be interpreted as a potential misadaptation to the stride pattern (7).

The zone of stride length reduction can be compared with the 400 m flat, which has a higher anaerobic component (14, 15, 21). However, stride length loss in the flat race also occurs around 250 m (31, 32). The data show that the number of stride pattern changes between the 8th and 9th hurdles explains a significant portion of the final number of steps variance. This is due to the importance of the second stride pattern change; higher-performing athletes generally maintain their hurdle rhythm throughout the race, adding only one more step between hurdles 6th and 8th, meanwhile LP and IP athletes predominantly performed 17 steps, instead of the 16 steps seen in the HP group between 8th and 9th hurdles. However, the hurdle clearance technique and their positioning require that decisions regarding a specific stride pattern be made differently than in the 400 meters flat. Athletes prefer performing motor actions with one side of the body over the other (33). Although 400-meter hurdles athletes must train both legs, they will have a dominant leg (7, 8). For this reason, the choice of stride pattern should also consider this preference. Additionally, the location of rhythmic changes influences performance, as attacking with the left leg allows athletes to run closer to the curve's inner lane (22) and a even stride pattern allows to attack all hurdles with athlete's dominant leg. However, an increase of two steps in an interval between hurdles will result in a sharp reduction in stride length, potentially causing a significant loss of speed due to an excessive reduction in stride length (22).

The effort distribution of athletes appears to have changed in recent years. Iskra et al. (5) found that the critical part of the race was between the 4th and 7th hurdles, where the first stride pattern change typically occurred. The study highlights a trend towards a “speed” strategy among athletes. The current world record holder Sydney McLaughlin is one athlete who has reduced her total step count in the years analysed in this study (34) and has an endurance strategy. Furthermore, the initial steps are also changing, with female athletes performing 14 steps in initial intervals (between H1-H5). The fatigue accumulated during the event is primarily due to acidosis (16) and both stride frequency and stride length are key factors for performance in the second half of the race (24, 25).

The stride pattern among top-level athletes varies significantly, even when the same athletes change structures in different qualifying rounds (8). On this study, only the 23% of the athletes maintained the same stride pattern throughout the study. It is important to note that many athletes showed significant improvement over the 3 years of the study. Research shows that both men and women use various stride patterns, as seen in the analysis of the 2019 World Championships in Doha, included in this study (27, 35). The data reveal 18 different stride patterns in the four championships analysed. The HP group athletes predominantly use an *n* + 1 pattern, and some athletes (24% of total performances) perform 14 steps between first and second hurdles. In the IP and LP groups, most athletes used an *n* + 1 + 1pattern (57% and 61% respectively). This is a shift from previous major championships, such as the 1993 World Championships in Stuttgart, where women typically performed two or three stride pattern changes (36) or the 2011 World Championships in Daegu, 37.5% of the finalists used an *n* + 1 pattern, while *n* + 1 + 1 was more common (62.5%) (6). Therefore, there is a trend that is changing and needs to be analysed, especially given the current situation where world records in the discipline are being broken and the overall level continues to rise. The study reveals that the *n* + 1 + 1 pattern, which was proposed as ideal for women according to Lindeman (22), has changed in recent years. Therefore, coaches need to take these changes into account and adjust their strategies accordingly.

Overall, the results of this study show significant changes in the pacing strategy of current female 400 mH athletes. The number of steps between hurdles is crucial for high performance in the 400 mH women. However, the strategy to achieve this performance can vary. The *n* + 1 pattern is more commonly used by higher-performing athletes, who predominantly start with 15 steps and change to 16 steps, as the number of steps in the final segment (between the 8th and 9th hurdles) appears to be crucial for 400 mH performance. Most athletes use the *n* + 1 or *n* + 1 + 1 pattern, with up to 18 different stride patterns identified, including odd patterns with changes that add two steps and allow the dominant leg to remain unchanged, as well as patterns where the number of steps is reduced and later increased. The first stride pattern change might be located between the 6th and 8th hurdles, anticipating it for athletes who add steps twice during the race. An appropriate pacing strategy is essential to optimise the 400 mH performance but it seems necessary to adjust the stride pattern to the individual characteristics of each athlete. Finally, this article is the first to study the stride patterns during this period (2019–2022), showing significant changes in the performance of women in the discipline. In the coming years, further research should be conducted to continue analysing performance in the 400 mH, especially following the breaking of the world record at the Paris 2024 Olympics.

## Data Availability

The raw data supporting the conclusions of this article will be made available by the authors, without undue reservation.
